# The growth pattern and microvasculature of pancreatic tumours induced with cultured carcinoma cells

**DOI:** 10.1054/bjoc.1999.1017

**Published:** 2000-02

**Authors:** K Mäkinen, S Loimas, P Nuutinen, M Eskelinen, E Alhava

**Affiliations:** Department of Surgery, Gene Therapy Unit, Kuopio University Hospital, Kuopio, FIN-70211, Finland; AI Virtanen Institute, University of Kuopio, Kuopio, FIN-70211, Finland

**Keywords:** animal, azaserine, disease model, microcirculation, pancreatic neoplasms, tumour cells

## Abstract

Pancreatic cancer is one of the most frustrating problems in gastroenterological surgery, since there is little we can do to improve the survival of patients with current treatment strategies. If one is to elucidate factors related to carcinogenesis, tumour biology, diagnostics and new treatment modalities of this malignant disease, then it is essential to develop a suitable animal model. In the present study we investigated rat pancreatic tumour growth after intrapancreatic injection of cultured pancreatic carcinoma cells (DSL-6A/C1), originally derived from an azaserine-induced tumour, as well as the features of tumour microcirculation using the microangiography technique. After intrapancreatic inoculation, tumours were detected in 64% of animals. A 1 cm^3^tumour volume was reached within 20 weeks after inoculation. The tumours were ductal adenocarcinomas. Larger tumours showed invasive growth and spreading into the surrounding tissues, mainly into spleen and peritoneum. Microangiography revealed that the pancreatic tumours had an irregular and scanty vessel network and there were avascular areas in the center of the tumour. The area between normal pancreas and the induced tumour had dense vascularization. Intrapancreatic tumour induction with cultured pancreatic carcinoma cells produced a solid and uniformly growing tumour in Lewis rats and it thus provides a possible model for pancreatic cancer studies. © 2000 Cancer Research Campaign


*British Journal of Cancer* (2000) **82**(4), 900–904
© 2000 Cancer Research Campaign
DOI: 10.1054/ bjoc.1999.1017, available online at http://www.idealibrary.com on IDEAL®

### The growth pattern and microvasculature of pancreatic tumours induced with cultured carcinoma cells

**K Mäkinen[1,2], S Loimas[2,3], P Nuutinen[1], M Eskelinen[1] and E Alhava[1,2]**

[1]Department of Surgery, [2]Gene Therapy Unit, Kuopio University Hospital, FIN-70211, Kuopio, Finland; [3]AI Virtanen Institute, University of Kuopio, FIN-70211, Kuopio, Finland

**Summary** Pancreatic cancer is one of the most frustrating problems in gastroenterological surgery, since there is little we can do to improve the survival of patients with current treatment strategies. If one is to elucidate factors related to carcinogenesis, tumour biology, diagnostics and new treatment modalities of this malignant disease, then it is essential to develop a suitable animal model. In the present study we investigated rat pancreatic tumour growth after intrapancreatic injection of cultured pancreatic carcinoma cells (DSL-6A/C1), originally derived from an azaserine-induced tumour, as well as the features of tumour microcirculation using the microangiography technique. After intrapancreatic inoculation, tumours were detected in 64% of animals. A 1 $cm^3$ tumour volume was reached within 20 weeks after inoculation. The tumours were ductal adenocarcinomas. Larger tumours showed invasive growth and spreading into the surrounding tissues, mainly into spleen and peritoneum. Microangiography revealed that the pancreatic tumours had an irregular and scanty vessel network and there were avascular areas in the center of the tumour. The area between normal pancreas and the induced tumour had dense vascularization. Intrapancreatic tumour induction with cultured pancreatic carcinoma cells produced a solid and uniformly growing tumour in Lewis rats and it thus provides a possible model for pancreatic cancer studies. © 2000 Cancer Research Campaign

**Keywords:** animal; azaserine; disease model; microcirculation; pancreatic neoplasms; tumour cells

Although some progress has been made in our understanding of pancreatic carcinogenesis, the cause of this highly malignant disease still remains unknown. It generally grows without presenting symptoms until late in its history and thus poses many problems for the diagnosing physician. A mere 5–22% of tumours are detected early enough to allow radical resection (Trede et al, 1990; Eskelinen et al, 1991; Warshaw and Fernandez-del Castillo, 1992; Janes et al, 1996), the only hope for cure. Although in recent selective studies, 5-year survival rates of up to 17–27% have been reported for curative resection of pancreatic cancer (Trede et al, 1990, 1998; Warshaw and Fernandez-del Castillo, 1992; Yeo et al, 1995), even those patients surviving pancreatic cancer for 5 years often die of local or metastatic late recurrence (Trede et al, 1998). It appears that the results of surgical treatment alone have reached a plateau in pancreatic cancer.

Since our understanding of the mechanisms of pancreatic carcinogenesis has broadened, new treatment strategies might be devised to blunt the biological aggressiveness of these tumours. A sound foundation for studies into tumour growth and progression as well as new treatment modalities in pancreatic cancer would be the development of a reproducible animal model. The two main rodent pancreatic cancer models, the nitrosoamine-induced hamster model and the azaserine-induced rat model have been extensively documented (Longnecker and Curphey, 1975; Pour et al, 1977, 1981, 1991; Longnecker et al, 1981, 1992; Pour, 1984; Egami et al, 1991; Pettengill et al, 1993; Mäkinen et al, 1998). In a preliminary study, we have reported that a tumour cell line (DSL-6A/C1), originally derived from an azaserine-induced primary tumour (Pettengill et al, 1993), produced a solid and uniformly growing tumour when it was transplanted subcutaneously (s.c.) (Mäkinen et al, 1998). However, there are no studies available on the growth pattern and microvasculature of rat intrapancreatic tumours induced with cultured carcinoma cells. In this study we describe the tumour take, growth and microvasculature, and subsequently evaluate the feasibility of this animal model for use in pancreatic cancer studies.

*Received 13 May 1999*
*Revised 17 August 1999*
*Accepted 26 August 1999*

*Correspondence to:* K Mäkinen

### MATERIALS AND METHODS

### Animals

Male Lewis rats ($n$ = 120) were obtained from Charles River Laboratories (Sulzfeld, Germany). For transplantation, 5-week-old rats (± 1 week) with initial weights of about 100 g were used. Studies and growth characteristics were based on five different transplantation series. The animals were kept under standard laboratory conditions, fed a chow diet, observed daily and autopsied if showing lethargy, ulcers, swelling or weight loss. All animal procedures were approved by the Animal Care and Use Committee of the University of Kuopio.

### Tumour cell line and transplantation

Tumour cell line DSL-6A/C1 (ATCC CRL-2132) was obtained from an acinar cell carcinoma (DSL-6) of rat pancreas (Pettengill et al, 1993). Cell culture and characteristics of s.c. transplanted tumours have been reported previously (Pettengill et al, 1993; Mäkinen et al, 1998). In brief, for pancreatic tumour induction,

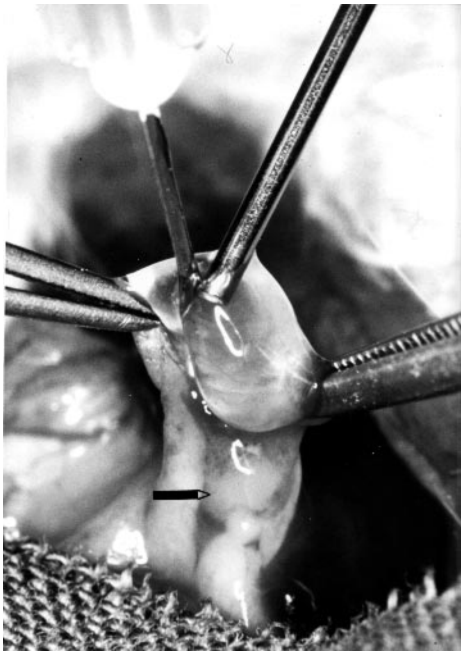

**Figure 1** Intrapancreatic tumour induction by injecting 2 million cultured DSL-6A/C1 carcinoma cells in 20 µl volume into the splenic lobe of Lewis rat pancreas. Injected carcinoma cells were seen in pancreas under the pancreatic capsule (arrow). Fibrin glue was applied over the injection area to prevent carcinoma cell spread before the needle was withdrawn

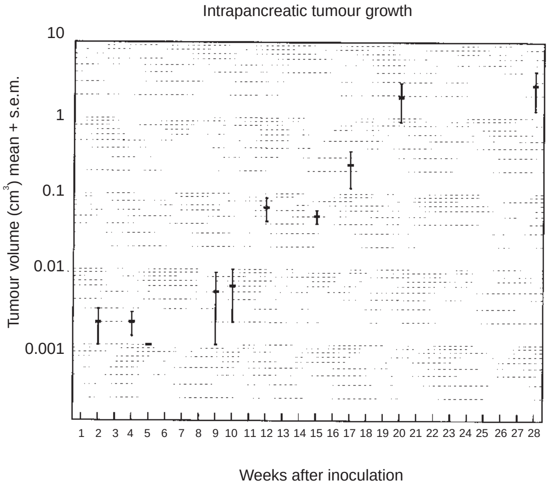

**Figure 2** The growth curve for Lewis rat intrapancreatic tumours. Each value is the mean ± s.e.m. of pancreatic tumour volumes measured from 5–10 animals

20 µl containing $2 \times 10^6$ carcinoma cells were injected through the pancreatic capsule into the pancreatic tissue at the splenic lobe (Figure 1) using Hamilton syringe (Hamilton Bonaduz AG, Switzerland) with 26-gauge needle and a stereotactic apparatus (Kopf, Germany). To prevent carcinoma cell spread after the injection, Tisseel fibrin glue (Immuno AG, Austria) was applied over the pancreas around the needle and after a 1-min delay the needle was slowly withdrawn.

### Tissue samples

At autopsy, 2–28 weeks after tumour induction, all tumours were removed, and their dimensions measured. The samples were then processed for light microscopy studies. The tumour volume was calculated in cubic centimeters. Abdomen and thoracic cavity were examined systematically throughout and all abnormal tissues were sampled and processed for microscopic examination.

### Microangiography

The method used has been described earlier (Kormano, 1967). Microangiography studies were carried out in untreated ($n = 2$) as well as in tumour-bearing animals ($n = 7$). Microangiography was performed by infusing 10% barium sulphate solution (Micropaque^R, Nicholas, France) into the abdominal aorta under 80–120 mmHg pressure until the pancreatic microvasculature was filled with contrast material. The whole pancreas was then removed and fixed in 4% buffered formaldehyde solution and embedded in paraffin. Five hundred-micrometer sections were cut for microangiography and adjacent 5 µm sections were cut and stained with haematoxylin and eosin (H&E) for histological studies. The microangiography slices were X-rayed with high resolution plates (Kodak^R), using a Siemens X-ray tube equipped with a tungsten anode and a beryllium window. The focus-to-film distance was 53 cm, and exposure values were 18 min, 40 kV and 20 mA. H&E samples were used for orientation and to confirm the borders of the tissue structures.

### Statistical analysis

In statistical analysis, the SPSS/PC+ program package (SPSS Inc, Chicago, II, USA) was used.

### RESULTS

Recovery after successful tumour cell inoculation was uneventful. The tumour take was 64% (77/120). At laparotomy the earliest palpable nodules were found 2 weeks after inoculation, but in some animals tumours became palpable only after 15 weeks. If there had been any technical problems in inoculation (i.e. carcinoma cell spreading) tumour tissue was found also around the peritoneum, mainly in the wound scars already after 2 weeks. The increase in tumour volume is shown in Figure 2. After 20 weeks the tumours usually had attained 1 $cm^3$ volume and in half of the experimental animals the tumours were still local. The others showed invasive growth around the peritoneum, gut mesentery, spleen, liver hilus, liver, kidneys and retroperitoneum. Ulcerative growth through the abdominal wall was found in the largest tumours. No distant metastases were found outside the abdomen. The consistency of tumours were hard. The cut surface was homogenous and pale, however, cystic changes were found in the largest (> 2.0 $cm^3$) tumours as well as the necrotic areas. Desmoplasia was a common finding. Tumour infiltrating lymphocytes and plasma cells as well as the granulocytes and giant cells of foreign type were found, although the inflammatory reaction was usually mild. All tumours were ductal adenocarcinomas showing usually moderate differentiation grade (Figure 3). No signs of perineural invasion were found.

*© 2000 Cancer Research Campaign*

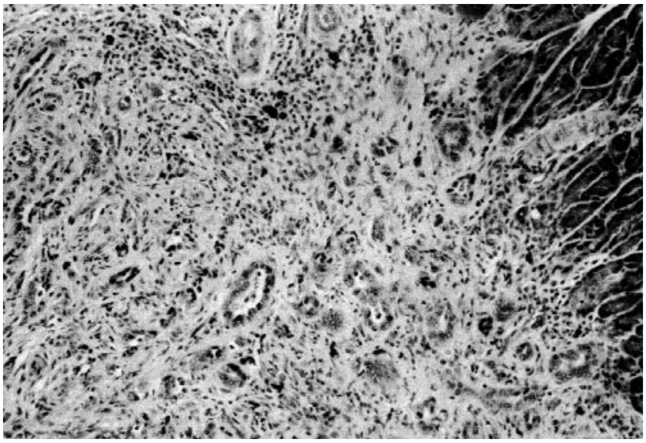

**Figure 3** Histologic appearance of pancreatic tumour taken 12 weeks after DSL-6A/Cl carcinoma cell inoculation exhibiting a moderately differentiated adenocarcinoma (right) growing infiltratively into normal pancreatic tissue (left). Scale bar = 100 μm

**Figure 4** Microangiography of the border between a pancreatic tumour (lower part) and normal pancreatic tissue (upper part) taken 15 weeks after carcinoma cell inoculation. Vessel network filled with contrast media invades into tumour tissue (**A**). Adjacent haematoxylin and eosin-stained section showing the border between induced carcinoma and normal pancreatic

The microvasculature, as well as arterioles and arteries, of pancreases were well filled with contrast medium. No ruptured vessels were found. Microangiography of normal rat pancreata showed that the vessels sprout like branches into a dense capillary network around pancreatic acini. The capillary structure was regular in shape and size. Pancreatic tumours exhibited deficits in their microcirculation. Tumour vessels were irregularly organized, slender, thin and straight ‘finger-like’ structures. There were avascular areas in the centre of the tumours. The peritumoural area showed dense neovascularization and vessel invasion (Figure 4).

### DISCUSSION

We have previously found that a tumour cell line (DSL-6A/C1) derived from azaserine-induced rat pancreatic tumour (Pettengill et al, 1993) produced a solid and uniformly growing tumour when it was transplanted s.c. into the Lewis rat (Mäkinen et al, 1998). However, our long-term strategy was to establish an intrapancreatic tumour model with a solid tumour nodule in the pancreas for pancreatic cancer studies, to be subjected to detailed examination in gene therapy studies.

In this experimental study we have demonstrated a reasonable tumour take and reproducible growth pattern after intrapancreatic tumour cell inoculation. In our series, 77 of 120 animals had pancreatic tumours when autopsied. A number of animals did not have palpable tumours until 15 weeks after cell inoculation. Since a few animals were autopsied before 15 weeks, those animals which did not have tumours at that time, would after all become tumour-bearing if they would have lived longer. Consequently, we believe that the actual tumour take was somewhat higher than 64%.

We conducted five different transplantation series and tumour take varied between these series although the cell culture, harvesting, animal age and inoculation techniques were similar. One explanation may be that tumourigenicity of the cell line varies. It is also possible that immunocompetent animals can destroy tumour cells to varying degrees. Although the inflammatory reaction within the tumours and in the peritumoural area was usually mild, some variation did exist. Interestingly, some animals that did not have tumours had inflammatory reaction with giant cells of foreign type, and some had also few atypical ductal cells as well as desmoplasia. In addition, some samples showed intense inflammation, as seen in the number of plasma cells and lymphocytes. This may be a reflection of immune rejection and tumour cell killing through graft-versus-host response. Consequently, if we had used immunodeficient animals the results might have been more favourable in regard to induction of tumours. However, the

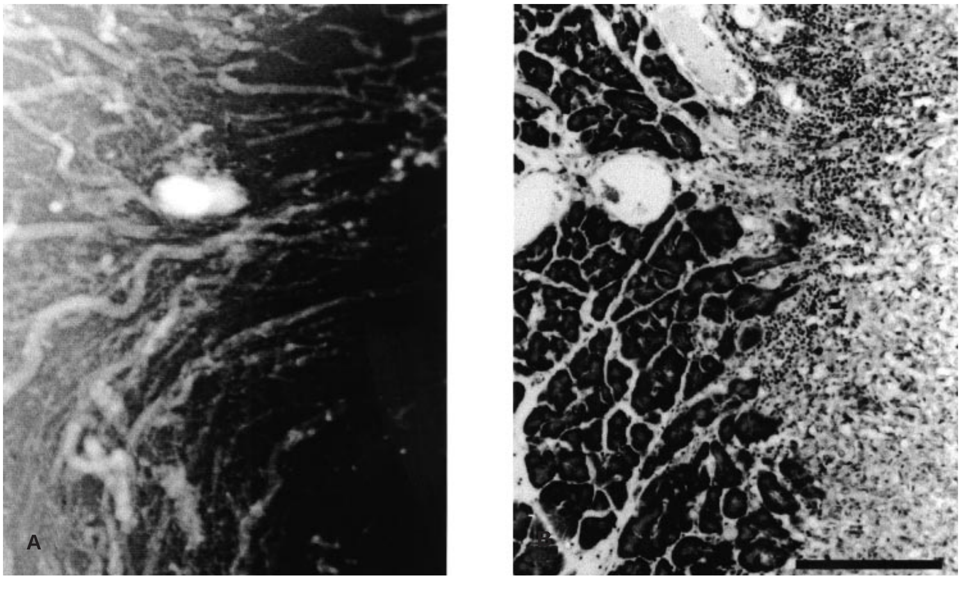

**Figure 4** Microangiography of the border between a pancreatic tumour (right) and normal pancreatic tissue (left) taken 15 weeks after carcinoma cell inoculation. Vessel network filled with contrast media invades into tumour tissue (**A**). Adjacent haematoxylin and eosin-stained section showing the border between induced carcinoma and normal pancreatic tissue (**B**). Scale bar = 100 μm

*© 2000 Cancer Research Campaign*

experiments then would have been much more complicated and the linkage of this model to human disease would have been diminished.

Intrapancreatic tumour induction with small fragments of rapidly growing azaserine-induced primary tumour was originally presented by Longnecker (1981). In this model pancreatic tumours grew rapidly, reaching 2–3 cm dimension in 3 weeks. This model has been adapted in intrapancreatically as well as in s.c.-induced tumours (Aprahamian et al, 1993; Karsently et al, 1993; Evrard et al, 1994). Although tumours induced with cultured cells grew more slowly, experimental tumours induced with a homogenous cell population may offer advantages with respect to similar biological behaviour. Our results concerning the phenotype and growth characteristics seem to confirm this hypothesis. Furthermore, a rodent tumour model with slowly progressing malignant tumour may provide better predictability when compared with models of very rapidly dividing and spreading rodent carcinoma cells.

In rats, azaserine treatment produces pancreatic tumours that arise from altered acinar cells (Longnecker, 1987). However, when these tumour cells were cultured and regrafted s.c. in Lewis rats, they produced duct-like carcinoma (Pettengill et al, 1993). As presented previously (Mäkinen et al, 1998) and in this study, intrapancreatic tumours induced with cultured cells also exhibited a ductal phenotype. The reason for this phenotypic plasticity has been discussed previously (Hall and Lemoine, 1993; Pettengill et al, 1993). Although DSL-6A/C1 cells produce ductal pancreatic carcinoma, there are some similarities with this tumour model and human acinar cell carcinoma of the pancreas as reported previously (Klimstra et al, 1992). The metastatic disease in their material was largely limited to the upper part of the abdomen. They also found recurrent tumour in the abdominal or chest wall and considered this as a consequence of carcinoma implantation in the surgical resection tracts. In our experimental model, the tumour spread was also limited to intra-abdominal organs and tumour dissemination to the abdominal wall around the scars was a common finding.

Experimental tumours showed deficits in their microvasculature with the shape of vessels being straight but their structure irregular. This is due, at least in part, to the necrosis and desmoplasia found in tumours. Previously, the microangiography technique has been used to analyse microcirculatory changes in experimental pancreatitis and hypovolemic shock (Nuutinen et al, 1986, 1988). These studies showed that poor filling of capillaries during haemorrhagic or necrotizing pancreatitis may be due to disruption of capillaries, vascular thrombosis or shunting of blood away from the capillary circulation. In addition, pancreatic ducts in the necrotic areas of severe acutic pancreatitis, as well as the ductal walls in chronic pancreatitis, showed a decrease in their vascularity (Pitkäranta et al, 1991). The dense vessel network in the peritumoural area was a particularly interesting finding of the present study. This may be a consequence of angiogenic switch, through changes in the balance between angiogenic activators and inhibitors (Hanahan and Folkman, 1996). Consequently, the tumour cells may either induce the production or even synthesize themselves mediators that stimulate angiogenesis, an essential factor for expansion of the tumour mass. This could be elucidated by analysing known angiogenic inducers, such as vascular endothelial growth factor and fibroblast growth factors before and during tumourigenesis. However, this is only one of many problems remaining to be answered.

We observed that a tumour cell line (DSL-6A/C1), derived from an azaserine-induced rat pancreatic tumour (DSL-6), produced a solid and uniformly growing tumour when transplanted intrapancreatically into the Lewis rat, providing a feasible model for pancreatic cancer studies.

### ACKNOWLEDGEMENTS

This work was financially supported by the EVO-fund financing system of Kuopio University Hospital, The Finnish Medical Foundation, The Finnish Medical Society Duodecim and the Savo Cancer Fund of the North-Savo Cancer Society.

### REFERENCES

Aprahamian M, Evrard S, Keller P, Tsuji M, Balboni G, Damge C and Marescaux J (1993) Distribution of pheophorbide A in normal tissues and in an experimental pancreatic cancer in rats. *Anticancer Drug Des* **8:** 101–114

Egami H, Tomioka T, Tempero M, Kay D and Pour PM (1991) Development of intrapancreatic transplantable model of pancreatic duct adenocarcinoma in Syrian golden hamsters. *Am J Pathol* **138:** 557–561

Eskelinen M, Lipponen P, Marin S, Haapasalo H, Mäkinen K, Ahtola H, Puittinen J, Nuutinen P and Alhava E (1991) Prognostic factors in human pancreatic cancer, with special reference to quantitative histology. *Scand J Gastroenterol* **26:** 483–490

Evrard S, Keller P, Hajri A, Balboni G, Mendoza-Burgos L, Damge C, Marescaux J and Aprahamian M (1994) Experimental pancreatic cancer in the rat treated by photodynamic therapy. *Br J Surg* **81:** 1185–1189

Hall PA and Lemoine NR (1993) Models of pancreatic cancer. *Cancer Surv* **16:** 135–155

Hanahan D and Folkman J (1996) Patterns and emerging mechanisms of angiogenic switch during tumorigenesis. *Cell* **86:** 353–364

Janes RH Jr, Niederhuber JE, Chmiel JS, Winchester DP, Ocwieja KC, Karnell LH, Clive RE and Menck HR (1996) National patterns of care for pancreatic cancer. *Ann Surg* **223:** 261–272

Karsently L, Hajri A, Aprahamian M, Garaud J-C, Doffoel M and Damge C (1993) Inhibition of growth of a transplanted rat pancreatic acinar carcinoma with CCK-8. *Pancreas* **8:** 204–211

Klimstra DS, Heffess CS, Oertel JE and Rosai J (1992) Acinar cell carcinoma of the pancreas. A clinicopathologic study of 28 cases. *Am J Surg Pathol* **16:** 815–837

Kormano M (1967) An angiographic study of the testicular vasculature in the postnatal rat. *Z Anat Entwicklungsgesch* **126:** 138–153

Longnecker DS (1981) Carcinoma of the pancreas in azaserine-treated rats. *Am J Pathol* **105:** 94–96

Longnecker DS (1987) The azaserine-induced model of pancreatic carcinogenesis in rats. In: *Experimental Pancreatic Carcinogenesis*, Scarpelli DG, Reddy JK and Longnecker DS (eds), pp. 117–130. CRC Press: Boca Raton, FL

Longnecker DS and Curphey TJ (1975) Adenocarcinoma of the pancreas in azaserine-treated rats. *Cancer Res* **35:** 2249–2258

Longnecker DS, Roebuck BD, Yager JD, Lilja HS and Siegmund B (1981) Pancreatic carcinoma in azaserine-treated rats: Induction, classification and dietary modulation of incidence. *Cancer* **47:** 1562–1572

Longnecker DS, Memoli V and Pettengill OS (1992) Recent results in animal models of pancreatic carcinoma: histogenesis of tumors. *Yale J Biol Med* **65:** 457–464.

Mäkinen K, Loimas S, Kosma VM, Wahlfors J, Pettengill OS, Longnecker DS, Johansson R and Alhava E (1998) Azaserine-induced rat pancreas tumor model with transplantable cultured cells. *Pancreas* **16:** 160–164

Nuutinen P, Kivisaari L, Standertskjöld-Nordenstam C-G, Lempinen M and Schröder T (1986) Microangiography of the pancreas in experimental hemorrhagic pancreatitis. *Eur J Radiol* **6:** 187–190

Nuutinen P, Kivisaari L and Schröder T (1988) Contrast-enhanced computed tomography and microangiography of the pancreas in acute human hemorrhagic/necrotizing pancreatitis. *Pancreas* **3:** 53–60

Pettengill OS, Faris RA, Bell RH Jr, Kuhlmann ET and Longnecker DS (1993) Derivation of ductlike cell lines from a transplantable acinar cell carcinoma of the rat pancreas. *Am J Pathol* **143:** 292–303

Pitkäranta P, Kivisaari L, Nordling S, Nuutinen P and Schröder T (1991) Vascular changes in pancreatic ducts and vessels in acute necrotizing, and in chronic pancreatitis in humans. *Int J Pancreatol* **8:** 13–22

© 2000 Cancer Research Campaign 

Pour PM (1984) Histogenesis of exocrine pancreatic cancer in the hamster model. *Envir Health Persp* **56:** 229–243
Pour P, Kruger FW, Althoff J and Mohr U (1977) A potent pancreatic carcinogen in Syrian hamsters: N-nitrosobis(2-oxopropyl)amine. *J Natl Cancer Inst* **58:** 1449–1453
Pour PM, Runge RG, Birt D, Gingell R, Lawson T, Nagel D, Wallcave L and Salmasi SZ (1981) Current knowledge of pancreatic carcinogenesis in the hamster and its relevance to the human disease. *Cancer* **47:** 1573–1587
Pour PM, Egami H and Takiyama Y (1991) Patterns of growth and metastases of induced pancreatic cancer in relation to the prognosis and its clinical implications. *Gastroenterology* **100:** 529–536
Trede M, Schwall G and Saeger H-D (1990) Survival after pancreaticoduodenectomy. *Ann Surg* **211:** 447–459
Trede M, Saeger HD, Schwall G and Rumstadt B (1998) Resection of pancreatic cancer – surgical achievements. *Langenbecks Arch Surg* **383:** 121–128
Warshaw AL and Fernandez-del Castillo C (1992) Pancreatic carcinoma. *N Engl J Med* **326:** 455–465
Yeo CJ, Cameron JL, Lillemoe KD, Sitzmann JV, Hruban RH, Goodman SN, Dooley WC, Coleman J and Pitt HA (1995) Pancreaticoduodenectomy for cancer of the head of the pancreas: 201 patients. *Ann Surg* **221:** 721–733

 *© 2000 Cancer Research Campaign*
